# Brucellar spondylitis is associated with disturbance in gut microbiota and histamine metabolism associated inflammation

**DOI:** 10.3389/fcimb.2026.1914654

**Published:** 2026-08-27

**Authors:** Zongjun Ma, Xiaoyan Bai, Jun Tian, Chenfei Yao, Zhijia Yan, Xiaolong Ma, Cunlin Zhang, Junbai Ma, Yuan Lin, Xiaoxia Zhang, Hao Wang

**Affiliations:** 1 Ningxia Key Laboratory of Infection and Immunity, School of Basic Medical Sciences, Ningxia Medical University, Yinchuan, China; 2 The First Clinical College, General Hospital, Ningxia Medical University, Yinchuan, China; 3 Department of Orthopedics, The Fourth People's Hospital of Ningxia Hui Autonomous Region, Yinchuan, China; 4 College of Traditional Chinese Medicine, Ningxia Medical University, Yinchuan, China

**Keywords:** brucellar spondylitis, gut microbiota, histamine metabolism, inflammation, SCFAs

## Abstract

**Background:**

The pathogenesis of brucellar spondylitis (BLS) has traditionally been considered to be primarily limited to local osteoarticular lesions. With the proposal of the “gut-spine axis” concept, the role of intestinal microecological dysbiosis in inflammatory spinal diseases has attracted in an increase of attention. The overactivated inflammatory cytokine network not only mediates bone destruction and intervertebral disc damage, but also forms a bidirectional interaction with gut microbiota dysbiosis through the “gut-spine axis,” collectively driving disease progression. However, the inflammatory mechanism by which gut microbiota participates in the pathological process of BLS remains largely unclear.

**Methods:**

This study recruited 20 BLS patients and 20 healthy donors. Multi-omics analysis including metagenomics, untargeted metabolomics, and targeted short-chain fatty acids (SCFAs) analysis, were used to compare the structural differences in gut microbiota between the two groups and screen for signature differential bacterial species. Plasma levels of histamine and histidine decarboxylase were measured by ELISA to clarify the role of differential histidine metabolic pathway in the disease. Additionally, plasma levels of lipopolysaccharide (LPS) and inflammatory cytokines (IL-1β, IL-6, IL-10, IL-17A, TNF-α) were detected by ELISA. The correlation between gut microbiota and inflammatory indicators was further analyzed.

**Results:**

Compared to the healthy control group, the α-diversity of the gut microbiota in BLS patients was significantly reduced, with the microbial community structure exhibiting increased homogeneity. Beta diversity analysis revealed significant differences, suggesting that disease progression is associated with an overall imbalance in the gut microbiota and the deterioration of its specific structural composition. At the phylum level, the abundances of *Actinomycetota*, *unclassified_d_Viruses*, and *Fusobacteriota* were significantly increased in the gut microbiota of BLS patients compared to the control group, while the abundances of *Bacillota* and *Pseudomonadota* were significantly decreased. Further analysis revealed that, compared to the control group, the generic abundance of *Enterococcus* was significantly increased, while the proportions of *Blautia, Faecalibacterium, Ruminococcus, Agathobacter, Roseburia, Clostridium, Eubacterium, Alistipes* and *Anaerobutyricum* were significantly decreased. At the species level, the abundances of *Enterococcus sp* and *Enterococcus-faecium* were increased, whereas *Blautia sp, Ruminococcus sp, Faecalibacterium sp, Faecalibacterium prausnitzii, Agathobacter rectalis, Eubacterium sp, Agathobacter sp*, and *Roseburia* sp were decreased. Furthermore, untargeted metabolomics revealed that metabolites were enriched in the histidine metabolic pathway, and the levels of SCFAs including butyrate, isobutyrate, valerate, and 4-methylvalerate in the intestinal contents were reduced in BLS. Functional KEGG profiling revealed that key KOs involved in butyrate synthesis (e.g., K00074, K00172, K01640) and transport were globally downregulated in the patient group, whereas histidine decarboxylase KOs (K01693, K11755, K19787) that convert histidine to pro-inflammatory histamine were significantly enriched. The loss of butyrate-producing symbionts led to SCFAs deficiency and mucosal barrier disruption, creating ecological niches for facultatively anaerobic Enterococcus, which further exacerbated local inflammation via proteolytic fermentation and histamine production. Compared with the control group, BLS patients showed decreased plasma levels of IL-10, while levels of IL-1β, IL-6, IL-17A, and TNF-α were increased, and LPS levels were elevated. In addition, significantly elevated plasma pro-inflammatory LPS levels in patients with BLS suggest disruption of intestinal integrity and permeability. Correlation analysis indicated a close relationship between gut microbiota and inflammation.

**Conclusion:**

BLS is associated with gut microbiota dysbiosis and alterations in microbial metabolites, which may be linked to inflammatory responses and histamine metabolism. The differential microbial taxa identified in this study could be developed into a stool-based non-invasive diagnostic panel to facilitate early differentiation of BLS from other spinal disorders. Furthermore, restoring gut microbial balance through probiotic supplementation or dietary modulation may represent a promising adjunctive strategy to enhance the efficacy of standard antibiotic therapy and reduce disease recurrence.

## Introduction

Brucellosis is a zoonotic infectious disease caused by bacteria of the genus Brucella. Unlike pyogenic spondylitis caused by nonspecific pyogenic bacteria, the major pathogenic species include B. melitensis, B. abortus, B. suis, and B. canis, among which B. melitensis is the most common pathogen responsible for human infections (World Health Organization, 2020). This disease can affect multiple organ systems, with osteoarticular involvement being the most common manifestation, with a reported prevalence ranging from 10% to 85%. particularly in the spine (He and Zhang, 2018), which occurs in 2% to 54% of cases. The lumbar spine is the most frequently affected site, with involvement rates ranging from 59.8% to 78.3% across different study populations (Feng et al., 2025). Known risk factors for the development of BLS include close contact with livestock, consumption of unpasteurized animal products, older age, delayed diagnosis, elevated erythrocyte sedimentation rate, and chronicity of disease. Factors associated with poor prognosis include delayed diagnosis, diabetes mellitus, paravertebral abscess, and neurological impairment (Spernovasilis et al., 2024). The disease is highly endemic in the Mediterranean basin, the Middle East, Central Asia, South America, and the pastoral regions of northern and western China, particularly Xinjiang, Inner Mongolia, and Ningxia (Wang et al., 2025; Wu et al., 2025). Although the overall mortality of BLS is relatively low, delayed or inappropriate treatment often leads to serious consequences. Therefore, elucidating the pathogenesis of BLS and exploring more effective therapeutic targets are of great theoretical and clinical importance.

Traditionally, the pathogenesis of BLS has been considered to be mainly restricted to local lesions in the spinal joints and surrounding tissues (World Health Organization, 2020). However, with the proposal of the “gut microbiota–bone axis” theory, increasing evidence indicates that intestinal microecological dysbiosis is closely associated with the occurrence and development of spinal inflammation (Na et al., 2021). Specifically, it has been well established that gut dysbiosis, characterized by increased *Enterobacteriaceae* and reduced microbial diversity, is frequently observed in autoimmune SpA/AS. This dysbiosis promotes systemic inflammation by increasing intestinal permeability and activating the IL-23/IL-17 axis (Farsadi et al., 2025). Moreover, to broaden the implication of dysbiosis in spine-related disorders, we have cited a recent Mendelian randomization study demonstrating that specific taxa (e.g., *Betaproteobacteria* and *Burkholderiales*) are causally associated with an elevated risk of septic arthritis (Bai et al., 2024). In parallel, we also note that in the field of infectious diseases, pulmonary tuberculosis is accompanied by gut dysbiosis and altered short-chain fatty acid metabolism, which may affect host susceptibility to Mycobacterium tuberculosis (Yu et al., 2023; Zhang et al., 2024).Furthermore, Short-chain fatty acids (SCFAs), as key metabolites of the gut microbiota, are key molecules linking the gut microbiota to bone metabolism (Ohlsson and Sjögren, 2015),which can promote osteoblast function via the insulin-like growth factor 1 (IGF-1) signaling pathway, suggesting that intestinal microecological dysbiosis is an important risk factor for abnormal bone metabolism (Lucas et al., 2018). Reduction of SCFAs compromises the intestinal barrier function, promoting the translocation of bacterial endotoxins (such as LPS) into the bloodstream and triggering systemic low-grade inflammation (Ticinesi et al., 2025). Moreover, the decrease in SCFAs directly modulates the balance between osteoclasts and osteoblasts through activating G protein-coupled receptors (such as GPR41,43, 109a) or inhibiting histone deacetylases (HDACs), ultimately disrupting bone homeostasis and exacerbating inflammatory erosion and loss in bone and joint diseases (Luo et al., 2024).In addition, Reshaping gut microbiota homeostasis contribute to alleviating inflammation in the treatment of various human chronic diseases, such as obesity, diabetes, polycystic ovary syndrome, atherosclerosis, alcoholic liver disease, and schizophrenia (Wang et al., 2020; Zhu et al., 2020; Guo et al., 2021; Li et al., 2022; Ren et al., 2022). Therefore, modulation of the gut microbiota may offer a novel therapeutic strategy for spinal health.

Inflammation is a core pathological process in the onset and progression of BLS. Following hematogenous dissemination to the vertebral body, *Brucella* triggers excessive release of inflammatory cytokines TNF-α, IL-6, and IL-17 by activating macrophage polarization toward the M1 phenotype and Toll-like receptor signaling pathways. This subsequently induces osteoclast activation via the RANKL signaling pathway (Luo et al., 2024), mediating vertebral bone destruction and intervertebral disc damage. Recent studies suggest a bidirectional interaction between gut microbiota dysbiosis and the local spinal inflammatory microenvironment, potentially through a “gut-spine axis,” collectively driving the chronic progression of the disease (Miyauchi et al., 2020). Therefore, elucidating the regulatory mechanisms of the inflammatory network and its association with the gut microbiota not only helps to reveal the pathogenesis of BLS, but also provides important directions for the discovery of novel biomarkers and targeted intervention strategies.

The present study was designed to use metagenomic and metabolomic to systematically analyze the composition and metabolites of the gut microbiota in BLS patients, which may lays a foundation for further investigation of the cause and control of BLS.

## Materials and methods

A total of 20 eligible patients and 20 healthy volunteers were enrolled in this study, all of whom were recruited from the Fourth People’s Hospital of NingXia during the period from July 2024 to July 2025,which is the only tertiary infectious disease specialist hospital in the region, with an annual patient volume of 8,708 cases in the infectious diseases department. Cases of BLS strictly met the inclusion criteria specified in the Expert Consensus on the Updated Guidelines for the Diagnosis of Human Brucellosis – China, 2019. This study was approved by the Ethics Committee of General Hospital of Ningxia Medical University (No.KYLL-2022-0859) and conducted in compliance with relevant guidelines and regulations. Written informed consent was obtained from all subjects prior to the study.

### Inclusion criteria for cases

Based on the patient’s clinical presentation, laboratory tests and imaging results, any positive result from a pathogen or serological test. Data include, but are not limited to: Clinical characteristics: history of livestock exposure; patients presented with fever (undulant fever), fatigue, hyperhidrosis, limited spinal mobility, and positive tenderness and percussion pain at the spinous processes and paraspinal areas of the affected segments. Laboratory examinations: elevated infectious indicators such as erythrocyte sedimentation rate (ESR) and C-reactive protein (CRP).

Bacterial culture: Peripheral venous blood samples were collected aseptically from each enrolled patient prior to antibiotic administration. Blood cultures were performed using an automated continuous-monitoring blood culture system (BACT/ALERT 3D, bioMérieux, France). A minimum of 8–10 mL of whole blood was collected from each patient and inoculated into paired aerobic and anaerobic blood culture bottles. All culture bottles were incubated at 35–37 °C for up to 30 days, with daily inspection for signs of growth. Upon positive signal, aliquots were sub cultured onto Columbia blood agar and chocolate agar plates and incubated at 35 °C in 5–10% CO_2_ for 48–72 hours. Suspected Brucella colonies were identified by Gram staining (gram-negative coccobacilli), oxidase and urease tests, and confirmed by MALDI-TOF mass spectrometry (Bruker Daltonics, Germany). Antimicrobial susceptibility testing was performed using the broth microdilution method according to the Clinical and Laboratory Standards Institute (CLSI) guidelines for slowly growing bacteria (CLSI M45). The antibiotics tested included doxycycline, rifampicin, ciprofloxacin, levofloxacin, streptomycin, and gentamicin. Brucella reference strain ATCC 54459 was used as the quality control strain. All manipulations of live Brucella cultures were conducted in a Class II biological safety cabinet within a BSL-2 laboratory, following standard biosafety protocols.

### Imaging examinations

X-ray showed mild bone destruction at the vertebral margins; CT revealed multiple small patchy, round or oval hypodense lesions at the vertebral margins, with hyperdense sclerotic bands, presenting the specific “lace-like vertebra” and “parrot beak” signs. MRI showed significantly low T1WI signal intensity in the affected vertebrae, localized bone destruction at the edges of the vertebrae adjacent to the involved intervertebral disc, hyperemia and edema of paraspinal soft tissues, as well as abnormal signals in the paraspinal region and psoas major muscle. Complete medical records, including admission records, discharge records, surgical records, and relevant auxiliary examinations. Exclusion criteria: Patients with other spinal infections (such as spinal tuberculosis, pyogenic spondylitis, and other infectious spinal diseases) and spinal malignant tumors were excluded.

### Inclusion criteria for healthy controls

Individuals who underwent health check-ups at the Fourth People’s Hospital of Ningxia Hui Autonomous Region during the period from July 2024 to July 2025;

1. Age- and sex-matched to the case group;
2. No clinical symptoms related to brucellosis, such as low back pain, fever, or night sweats;
3. Negative Brucella serological tests;
4. Voluntary provision of written informed consent.

### Exclusion criteria for healthy controls

1. Previous history of brucellosis;
2. History of close contact with livestock (cattle, sheep, etc.) or consumption of unpasteurized dairy products;
3. Presence of other spinal diseases, including spinal tuberculosis, pyogenic spondylitis, or spinal tumors;
4. Presence of severe comorbidities involving the heart, liver, kidney, or autoimmune diseases;
5. Pregnant or lactating women;
6. Contraindications to MRI examination (e.g., metallic implants, claustrophobia).

These criteria are consistent with established practices in previous studies on BLS. Multiple studies have demonstrated that healthy controls in case-control studies of BLS should have no history of Brucella infection and negative serological test results. Additionally, other spinal pathologies such as spinal tuberculosis and pyogenic spondylitis should be excluded to avoid confounding effects. All healthy controls underwent detailed medical history taking, physical examination, and Brucella serological screening prior to enrollment to ensure compliance with the above criteria.

### Clinical measurements

The following data were collected from the patients: gender, age, ethnicity, place of residence, occupation, history of smoking and heavy drinking, history of livestock exposure, clinical manifestations including fever, hyperhidrosis, fatigue, and weight loss, initial laboratory examinations on admission including white blood cell count, neutrophil count, lymphocyte count, C-reactive protein (CRP), erythrocyte sedimentation rate (ESR), Brucella agglutination test, imaging examinations (including X-ray, CT, and MRI), and treatment regimens.

### Metagenomic analysis

Fresh fecal samples were collected from patients and healthy volunteers, immediately frozen in liquid nitrogen, and stored at -80 °C.

DNA extraction: 0.2g of stool material was used to extract total genomic DNA with the TIANMicrobe Magnetic Envir-DNA Kit 4 (TIANGEN, Beijing, China) according to manufacturer’s instructions. Concentration and purity of extracted DNA was determined with SynergyHTX and NanoDrop2000, respectively. DNA quality was checked on 1% agarose gel.

Metagenomic sequencing: DNA extract was fragmented to an average size of about 350 bp using SCIENTZ08-III Non-Contact Ultrasonic Cell Homogenizer (Ningbo Scientz Biotechnology Co. Ltd. Zhejiang, China) for paired-end library construction. Paired-end library was constructed using NEXTFLEX Rapid DNA-Seq (Bioo Scientific, Austin, TX, USA). Paired-end sequencing was performed on Illumina NovaSeq™ X Plus (Illumina Inc., San Diego, CA, USA) at Majorbio Bio-Pharm Technology Co., Ltd. (Shanghai, China) using NovaSeq X Series 25B Reagent Kit according to the manufacturer’s instructions (www.illumina.com). The metagenomic sequencing data associated with this project have been deposited in the NCBI Short Read Archive database (No. PRJNA1464200).

Processing of metagenome sequencing data: The data were analyzed on the free online platform of Majorbio Cloud Platform (www.majorbio.com). Briefly, the raw sequencing reads were trimmed of adapters, and low-quality reads (length<50 bp or with average quality value <20) were removed by fastp (https://github.com/OpenGene/fastp, version 0.23.0). Reads were aligned to the human genome by BWA (Li and Durbin, 2009) (http://bio-bwa.sourceforge.net, version 0.7.17) and any hit associated with the reads and their mated reads were removed. The quality-filtered data were assembled using MEGAHIT (Li et al., 2015) (https://github.com/voutcn/megahit, version 1.1.2). Contigs with a length ≥ 300 bp were selected as the final assembling result. Open reading frames (ORFs) from each assembled contigs were predicted using Prodigal (Fu et al., 2012) (https://github.com/hyattpd/Prodigal, version2.6.3) and a length ≥ 100 bp ORFs were retrieved. A non-redundant gene catalog was constructed using CD-HIT (https://github.com/weizhongli/cdhit/releases, version 4.6.1) with 90% sequence identity and 90% coverage. Gene abundance for a certain sample was estimated by SOAPaligner (Li et al., 2008) with 95% identity.

Taxonomic and functional annotation: The best-hit taxonomy of non-redundant genes was obtained by aligning them against the NCBI NR database by DIAMOND (Buchfink et al., 2015) (http://ab.inf.uni-tuebingen.de/software/diamond/,version 2.0.13) with an e-value cutoff of 1e-5. Similarly, the functional annotation (GO, KEGG, eggNOG, CAZy, CARD, PHI) of non-redundant genes was obtained. Based on the taxonomic and functional annotation and the abundance profile of non-redundant genes, the differential analysis was carried out at each taxonomic, functional, or gene-wise level by Kruskal-Wallis test.

### Fecal SCFAs quantification by gas chromatography-mass spectrometer

The quantification analysis of fecal SCFAs was performed using an Agilent 7890A, gas chromatography coupled with Agilent 5975C mass spectrometric detector (Agilent Technologies, United States) equipped with an HP-5MS column (0.25 × 30 mm, 0.25 µm particle size) (Suzhou Bionovogene Co, Ltd) as described previously. Helium was used as a carrier gas at a constant flow rate of 1 ml/min. The initial oven temperature was held at 60 °C for 5 min, ramped to 250 °C at a rate of 10 °C/min, and finally held at this temperature for 5 min. The temperatures of the front inlet, transfer line, and electron impact (EI) ion source were set as 280, 250, and 230 °C, respectively. Data handling was performed with an Agilent MSD ChemStation (E.02.00.493, Agilent Technologies, Inc., United States).

### Untargeted metabolomics

Collected feces were stored in liquid nitrogen at -80 °C. The homogenate was thoroughly resuspended by vortexing in pre-chilled 80% methanol. Samples were incubated on ice for 5 min, then centrifuged at 15,000×g and 4 °C for 20 min. The samples were incubated on ice for 5 min and then were centrifuged at 15,000 g, 4 °C for 20 min. Some of supernatant was diluted to final concentration containing 53% methanol by LC-MS grade water. The samples were subsequently transferred to a fresh Eppendorf tube and then were centrifuged at 15000×g, 4 °C for 20 min. Finally, the supernatant was injected into the LC-MS/MS system analysis.

### Determination of histamine and histidine decarboxylase in the histidine metabolism pathway

Quantitative detection of histidine decarboxylase (HDC) and histamine (HIS) levels was performed using commercial ELISA kits (Human Histidine Decarboxylase/Histamine ELISA Kit, Nanjing Boyan Biotechnology Co., Ltd., Nanjing, China) according to the manufacturer’s instructions. Samples were added to microplates pre-coated with anti-HDC antibody, incubated with biotinylated detection antibody and HRP-conjugated streptavidin, followed by addition of TMB substrate for color development. The absorbance was measured at 450 nm, and concentrations of HDC and HIS were determined from standard curves.

### Determination of plasma inflammatory factors

Plasma inflammatory cytokines, including IL-10, IL-17A, TNF-α, IL-1β and IL-6, were measured according to the manufacturer’s instructions (Jianglai Biotechnology Co., Ltd., Shanghai, China). The sensitivities for IL-1β, IL-6, IL-10, IL-17A and TNF-α were 0.1, 1.0, 0.1, 0.1 and 1.0 pg/mL, respectively. Optical density was measured at 450 nm within 15 min using an automated microplate reader (Thermo Scientific, United States).

### Plasma LPS assay

As previously described, lipopolysaccharide (LPS) was detected using a commercial kit (Jianglai Biotechnology Co., Ltd., Shanghai, China) according to the manufacturer’s instructions.

### Statistical analysis

Data analysis was performed using GraphPad Prism software (version 8). Data were presented as mean ± standard error of the mean (SEM). Normality and lognormality tests were performed prior to parametric analysis using an unpaired Student’s t-test. Correlation analysis was performed using Spearman analysis. A *P*-value < 0.05 was considered statistically significant.

## Results

### Clinical features of BLS

The clinical characteristics of the participants are summarized in **Table 1**. In this study, we analyzed the samples and clinical data of 40 individuals. The results showed significant differences in white blood cell count (*P* < 0.001), lymphocyte count (*P* < 0.001), C-reactive protein (*P* = 0.0015), and erythrocyte sedimentation rate (*P* < 0.001), while no significant difference was observed in neutrophil count (*P >*0.05). There were no significant differences in age or body weight between the two groups (*P*>0.05).

**Table 1:** Distribution of clinical characteristics of the participants.

Participants, n=40	NC=20	BLS=20	*P*-value
Basic characteristics			
Age	50.65 ± 7.9	54.65 ± 13.86	0.2692
Body weight	67.26 ± 5.27	67.26 ± 5.27	>0.9999
Laboratory findings			
White blood cell count****	5.3 ± 1.75	10.6 ± 4.39	<0.0001
Neutrophil count	5.55 ± 10.85	7.18 ± 10.48	0.6320
Lymphocyte count****	3.09 ± 1.47	29.07 ± 9.63	<0.0001
C-reactive protein**	7.46 ± 2.26	26.24 ± 24.41	0.0015
Erythrocyte sedimentation rate****	5.95 ± 2.11	42.6 ± 23.39	<0.0001

**Figure 1:**
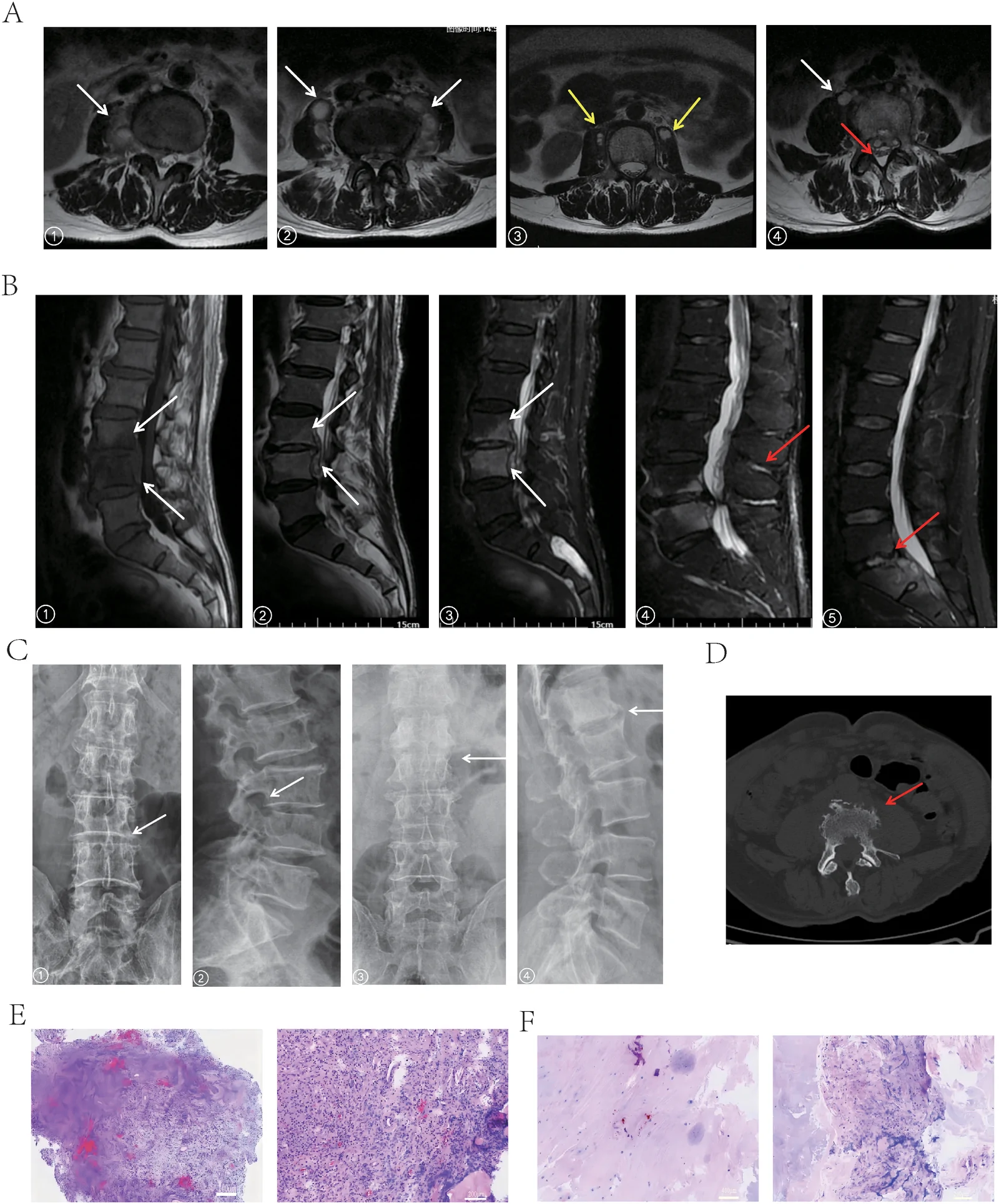
Clinical imaging and histopathological features of brucellar spondylitis. **(A)**. Axial spinal MRI. Paravetebral and perivertebral abscesses (white arrows), psoas abscess (yellow arrow), and intraspinal abscess (red arrow). **(B)**. Sagittal spinal MRI. ①. L3/4 vertebral bodies showing hypointense or isointense signal on T1-weighted imaging; ②. L3/4 vertebral bodies demonstrating hyperintense signal on T2-weighted imaging; ③. L3/4 vertebral bodies with hyperintense signal on fat-suppressed T2-weighted imaging; ④. hyperintense signal in the interspinous ligament at L4/5 on fat-suppressed imaging, suggesting interspinous ligament infection; ⑤. hyperintense signal in the L5/S1 intervertebral disc on fat-suppressed imaging, indicating discitis. **(C)**. Anteroposterior and lateral digital radiography (DR) of the spine. ① and ② narrowing of the L3/4 intervertebral space; ③ and ④ narrowing of the L1/2 intervertebral space, associated with a “parrot-beak” sign and anterior vertebral bony bridging. **(D)**. Axial spinal CT. **(E, F)**. Hematoxylin and eosin (HE) staining of bone tissue. (10X and 20X).

Comparison of clinical variables among groups. A ttest was used to assess statistical significance. Data are presented as mean ± standard deviation or as sample size per group. Significant differences: **P < 0.01, ****P < 0.0001 versus the NC group.

we systematically reviewed the lumbar MRI, DR and ct of all 20 patients who met the diagnostic criteria for BLS. mainly manifested as Localizing bone destruction appears as worm-eaten appearance, while diffuse bone destruction manifests as patchy or large-area bone defect shadows. Intervertebral space changes include narrowing and disappearance. Paravertebral abscess: Soft tissue shadows with ill-defined boundaries are seen around the vertebral bone destruction area. Periosteal changes: Periosteal proliferation, thickening and ossification at the vertebral margins, with osteophytes showing “beak-like”, “lip-like” or “cauliflower-like” signs. Osteophytes between adjacent vertebrae are connected to form “bone bridges”. Ligament changes: Mainly involving the interspinous ligament, posterior longitudinal ligament and anterior longitudinal ligament. The bone destruction and associated infectious lesions were classified into the following five major imaging patterns, and the number of patients in each pattern was reported:

1. Isolated disc space infection (signal abnormalities in the disc and adjacent endplates without paravertebral, vertebral, or spinal canal involvement) (**Figure 1B**): 8 patients (40%).
2. Disc space infection with paravertebral abscess (disc infection accompanied by paravertebral soft tissue abscess, e.g., psoas or perivertebral abscess) (**Figures 1A, B**): 5 patients (25%).
3. Disc space infection with vertebral body destruction (disc infection with adjacent vertebral bone erosion, moth-eaten appearance, or signal changes) (**Figures 1B, D**): 4 patients (20%).
4. Disc space infection with vertebral body destruction and paravertebral abscess (combined vertebral bone destruction and paravertebral abscess) (**Figures 1A, C**): 2 patients (10%).
5. Disc space infection with vertebral body destruction, paravertebral abscess, and intraspinal abscess (the most severe form, involving the spinal canal) (**Figures 1A-C**): 1 patient (5%).

These categories covered all cases with bone-destruction-related imaging changes. Other ancillary findings (e.g., high signal on fat-suppressed images in the interspinous ligaments, high signal in the intervertebral disc, disc space narrowing, parrot-beak sign, and osteophyte formation) were also observed within the corresponding groups but were not used as primary classification criteria.

Hematoxylin-eosin (HE) staining of intervertebral cartilage tissue shows chondrocyte degeneration and necrosis, chronic inflammatory cell infiltration, and non-caseating granuloma formation (**Figures 1E, F**).

### Patients with BLS exhibited dysbiosis of gut microbiota

**Figure 2:**
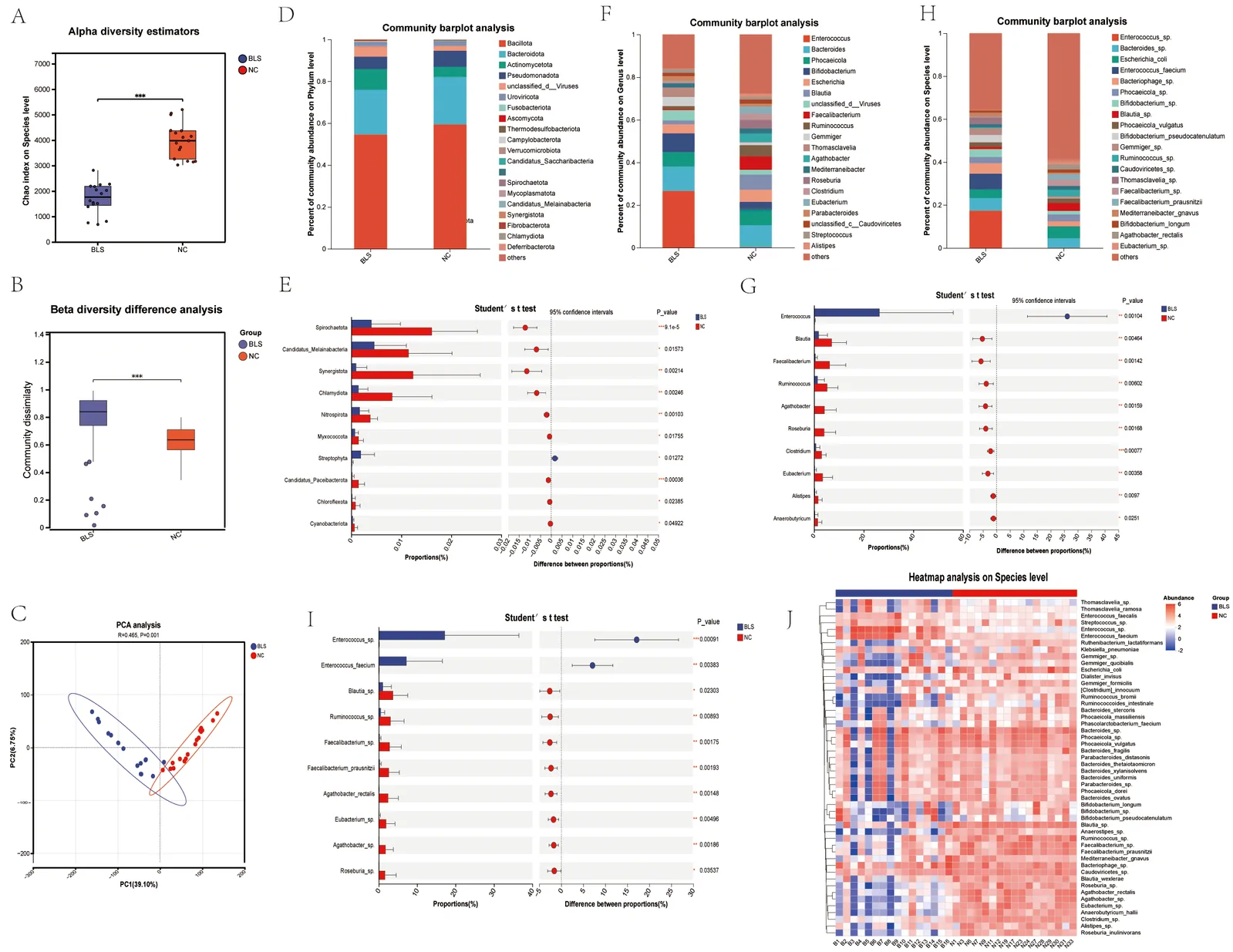
Alterations in the intestinal microbiome composition in patients with BLS compared to NC. **(A)** Alpha diversity analysis. **(B, C)** Beta diversity and principal component analysis (PCA). **(D, F, H)** Stacked bar plots of relative abundance. These panels show the taxonomic composition and mean relative abundances at the phylum **(D)**, genus **(F)**, and species **(H)** levels, respectively. For each group, the top 10 most abundant taxa are displayed. **(E, G, I)** Differential abundance analysis. Differentially abundant taxa at the phylum **(E)**, genus **(G)**, and species **(I)** levels were identified using Linear Discriminant Analysis (LDA) Effect Size (LEfSe). The LDA scores (log10) reflect the effect size and biological relevance of each taxon. **(J)** Clustering heatmap. Each row represents a species and each column represents a sample. The NC group is shown in red and the BLS group in blue. Data are presented as mean ± SEM, with n = 16 per group. Comparisons between the two groups were performed using the Wilcoxon rank-sum test. Significance levels are denoted as *P < 0.05, **P < 0.01, ***P < 0.001, and *P* < 0.0001 versus the NC group.

To further investigate the specific differences in the microbiota between the NC group and the BLS group, we performed additional analyses. Alpha diversity gradually decreased from the NC group to the BLS group with disease progression, indicating that the disease was accompanied by remodeling of the overall microbial structure. The microbial richness in the BLS group was significantly reduced and tended to be simplified (**Figure 2A**). β-diversity analysis also revealed significant differences among the groups (*P* < 0.001), indicating that disease occurrence is associated with disruption of the overall microbial balance and deterioration of specific community structures (**Figure 2B**). PCA analysis indicated that the gut microbiota samples from BLS patients and healthy individuals were concentrated in specific quadrants and clearly separated, further confirming that the microbiota undergoes progressive and significant changes during the progression of BLS (**Figure 2C**). Next, the differences in gut microbiota at the phylum level among different groups were analyzed and compared the microbial composition. *Bacillota* and *Bacteroidetes* constituted the dominant phyla in all groups. The gut microbiota of the two groups exhibited distinct clustering patterns at the phylum level. In the control group, *Bacillota, Bacteroidota, Actinomycetota* and *Pseudomonadota* were relatively abundant. Compared with the control group, the relative abundances of *Actinomycetota* and *unclassified_d_Viruses* were significantly increased in the BLS group, accompanied by an elevated abundance of *Fusobacteriota.* In contrast, the abundances of *Bacillota* and *Pseudomonadota* were markedly decreased (**Figures 2D, E**). Similarly, a study of patients with axial spondyloarthritis (axSpA) complicated by ulcerative colitis found that the axSpA group had decreased *Bacillota* and *Faecalibacterium*, and increased *Proteobacteria* and *Escherichia_Shigella* (Zhangni et al., 2024). A systematic review of 28 studies confirmed that SpA patients show increased *Proteobacteria* and *Enterobacteriaceae*, and decreased *Bacteroidetes* and *Akkermansia* (Wang et al., 2025). In tuberculosis, studies have shown that TB patients exhibit decreased *Bacillota* and *Proteobacteria* and increased *Actinobacteria* and *Bacteroidetes* (Chai et al., 2025), with Mendelian randomization studies further confirming that *Bacillota* and *Proteobacteria* are associated with TB risk (Wu et al., 2025).These consistent patterns across different types of spondylitis suggest that reduced *Bacillota* and expansion of *Pseudomonadota* may represent a shared gut microbial signature of spinal inflammation, regardless of whether the etiology is autoimmune or infectious. Further analysis of inter-group differences at the genus and species levels revealed that, compared to the control group, the abundance of *Enterococcus* was significantly increased at the genus level, while the proportions of *Blautia*, *Faecalibacterium*, *Ruminococcus*, *Agathobacter*, *Roseburia*, *Clostridium*, *Eubacterium*, *Alistipes* and *Anaerobutyricum* were Substantially decreased (**Figures 2F, G**). At the species level, the abundances of *Enterococcus* and *Enterococcus-faecium* were increased, whereas those of *Blautia*, *Ruminococcus*, *Faecalibacterium*, *Faecalibacterium prausnitzii*, *Agathobacter rectalis*, *Eubacterium*, *Agathobacter*, and *Roseburia* were decreased (**Figures 2H, I**). A clustered heatmap of species abundances showed that clustered species varied substantially among groups (**Figure 2J**).

### KEGG functional association analysis of differential microbiota with SCFAs synthesis and histidine metabolism pathways

**Figure 3:**
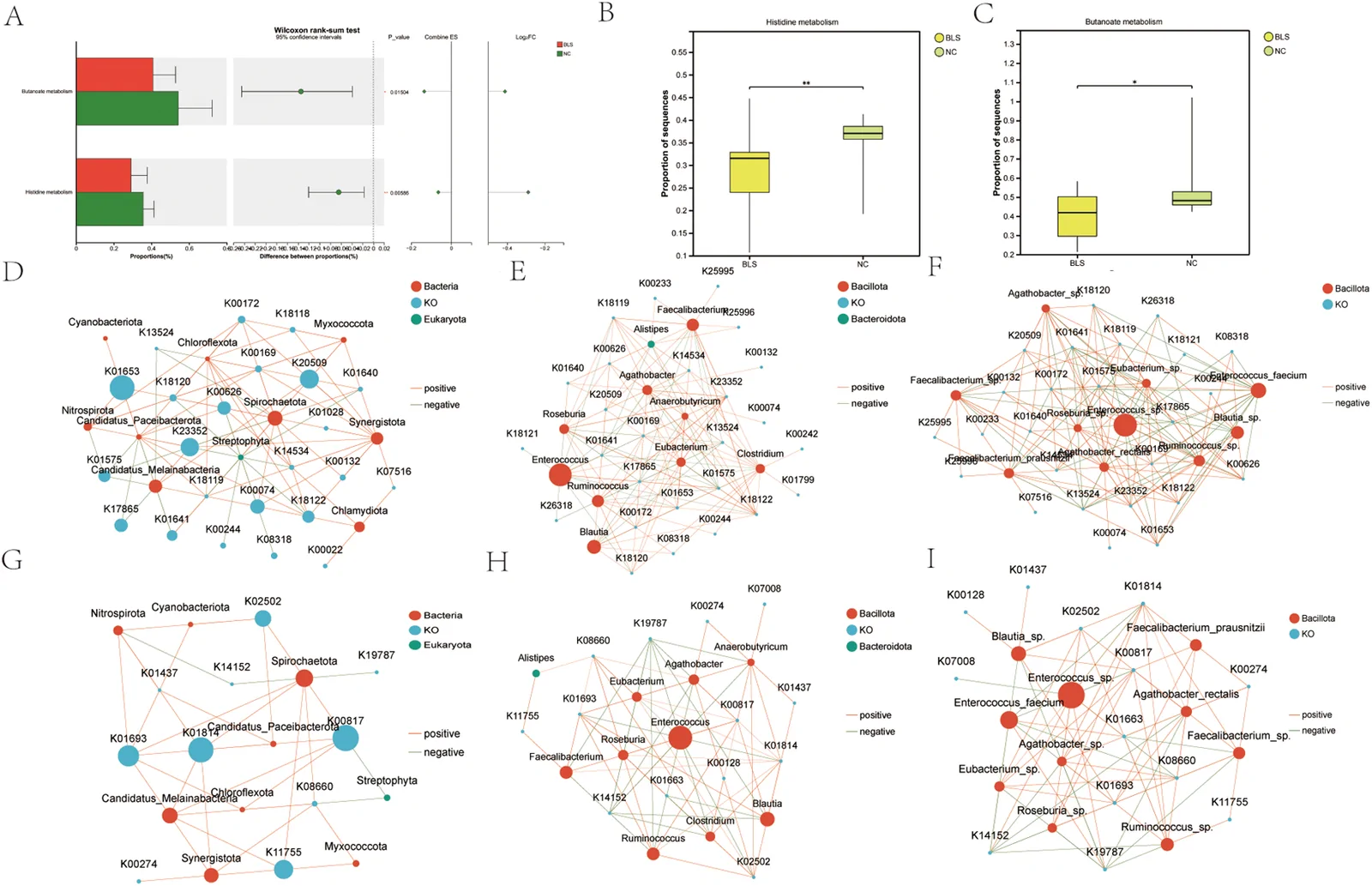
KEGG functional association analysis of differential microbiota with SCFA synthesis and histidine metabolism pathways. **(A)**. Differential analysis of KEGG functional profiles. **(B)**. Boxplot of the histidine metabolism pathway. **(C)**. Boxplot of the butyrate (butanoate) metabolism pathway. **(D)**. Association analysis between phylum-level taxa and the butyrate metabolism pathway. **(E)**. Association analysis between genus-level taxa and the butyrate metabolism pathway. **(F)**. Association analysis between species-level taxa and the butyrate metabolism pathway. **(G)**. Association analysis between phylum-level taxa and the histidine metabolism pathway. **(H)**. Association analysis between genus-level taxa and the histidine metabolism pathway. **(I)**. Association analysis between species-level taxa and the histidine metabolism pathway. Data are presented as mean ± SEM, with n = 16 per group. Significance levels are denoted as *P < 0.05, **P < 0.01 versus the NC group.

To elucidate the metabolic molecular mechanisms underlying differential gut microbiota in patients with BLS, we integrated taxonomic annotations at the phylum, genus and species levels together with KEGG KO functional gene profiles of two pathways (SCFAs synthesis and histidine metabolism) to perform a linkage analysis between differential microbial taxa and metabolic functions, addressing the limitation of merely listing altered microbes without functional evidence (**Figures 3A-C**).

At the phylum level, healthy intestines were enriched with anaerobic symbiotic phyla including *Candidatus_Melainabacteria* and *Chloroflexota*. The phylum *Bacillota*, which harbors core SCFAs-producing microbes, fell under these anaerobic groups. In contrast, the abundance of such anaerobic symbiotic phyla was markedly reduced in BLS patients, accompanied by massive enrichment of Gram-negative pro-inflammatory phyla such as *Chlamydiota, Spirochaetota* and *Synergistota* (**Figure 3D**).

Genus-level analysis further verified that canonical butyrate-producing genera including *Faecalibacterium*, *Roseburia*, *Blautia* and *Agathobacter* were significantly depleted in the patient group. These Gram-positive genera carry a full set of key KOs involved in butyrate synthesis (K00074, K00172, K01640, K01641, K00244, K0008318) and express functional genes K01437 and K02502 that maintain histidine homeostasis (**Figure 3H**). The reduction of these genera directly led to global functional exhaustion of the SCFAs synthesis pathway and impaired physiological histidine catabolism. Species-level microbial distribution was consistent with the patterns observed at the genus and phylum levels; the abundance of core anti-inflammatory strains *Faecalibacterium prausnitzii* and *Agathobacter rectalis* decreased drastically, resulting in a profound loss of intestinal SCFAs synthetic capacity.

On the contrary, the opportunistic pathogen *Enterococcus* (including *Enterococcus* sp. and *Enterococcus faecium*) underwent excessive proliferation in the gut of BLS patients. Functional annotation revealed that *Enterococcus* lacks a complete butyrate synthesis pathway and only encodes a small number of partial components for weak SCFAs production. Meanwhile, it is enriched with key histidine decarboxylase KOs (K01693, K11755, K19787) that catalyze the massive conversion of histidine into pro-inflammatory histamine (**Figures 3G, H**). The depletion of butyrate-producing symbionts causes butyrate deficiency and disrupted intestinal mucosal barrier, alongside a disturbed anaerobic gut microenvironment that generates favorable colonization niches for facultatively anaerobic *Enterococcus*. Relying on proteolytic fermentation and histamine synthesis, *Enterococcus* occupies the vacant ecological niches and further exacerbates local intestinal inflammation.

Global KEGG functional profiling demonstrated that KOs responsible for carbon source utilization, butyrate synthesis and product transport within the butanoate metabolism pathway (K13524, K18118–K18122, K20509, K23352) were generally less abundant in the BLS group (**Figures 3D-F**), whereas KOs related to pro-inflammatory histidine metabolism were significantly enriched (**Figures 3G-I**). The consistent variation trends of microbial community structure at three taxonomic ranks and functional genes of the two metabolic pathways mutually validated each other. Dysregulated SCFAs and histidine metabolism synergistically drive chronic spinal inflammation via the gut-spine axis.

### Levels of gut microbial SCFAs including butyric acid, isobutyric acid, valeric acid, and 4-methylvaleric acid were weakened in patients with BLS

**Figure 4:**
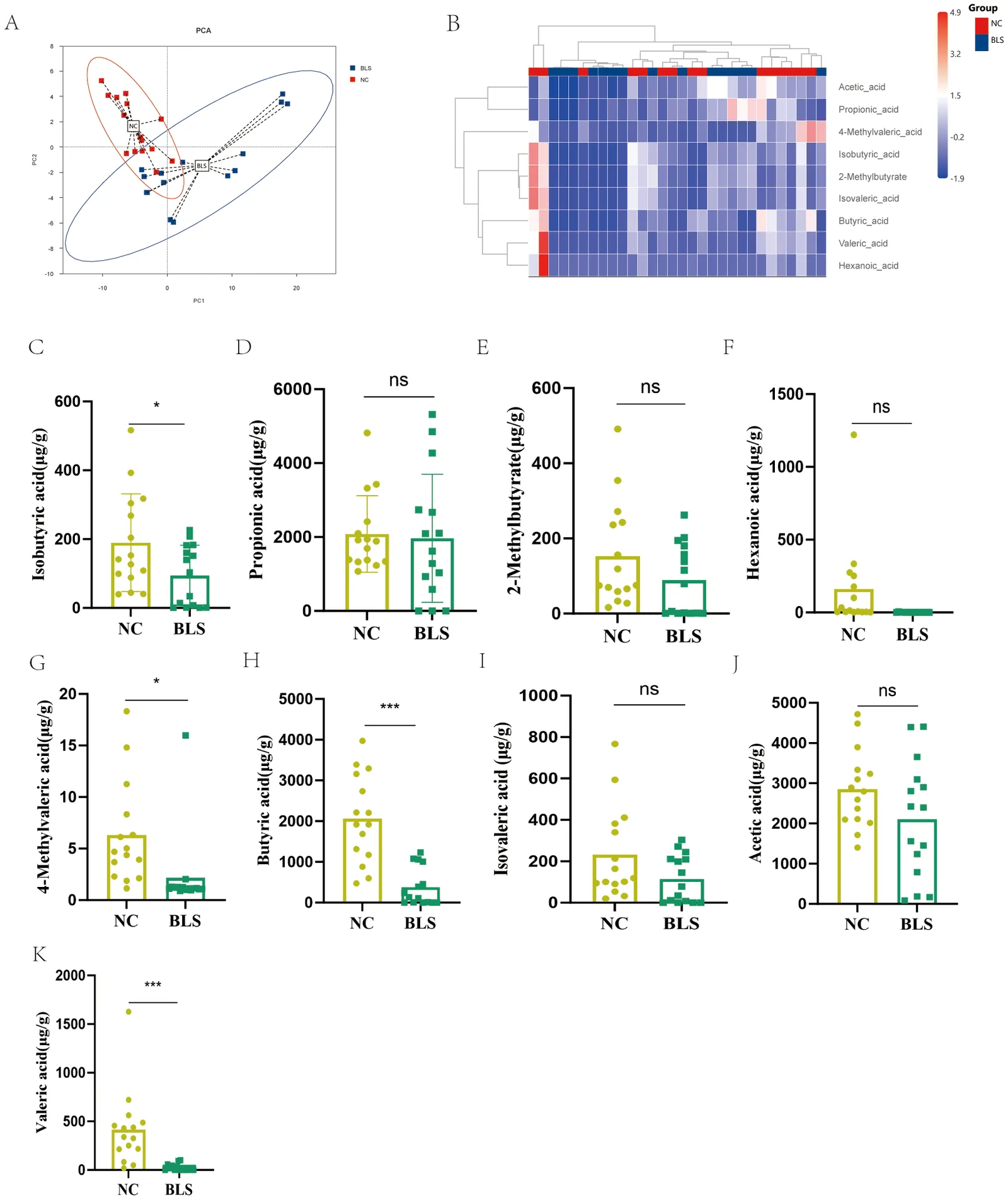
The contents of butyric acid, isobutyric acid, valeric acid and 4-methylvaleric acid in the intestinal contents of clinical patients with brucellar spondylitis were all decreased. **(A)**. PCA. **(B)**. Cluster heatmap. **(C–K)**. Detection of differential SCFAs. Data are presented as mean ± SEM, with n = 15 per group. Significance levels are denoted as *P < 0.05, ***P < 0.001 versus the NC group.

SCFAs, crucial metabolites derived from gut microbiota, can notably affect intestinal barrier function. Therefore, in this study, gas chromatography-mass spectrometry (GC-MS) was used to detect SCFAs in patients and healthy volunteers’ feces, mainly including acetic acid, propionic acid, butyric acid, valeric acid, isovaleric acid, 2-methylbutyric acid, isobutyric acid, 2-methylvaleric acid, 3-methylvaleric acid, hexanoic acid, and isohexanoic acid. PCA analysis generally reflects the overall metabolic differences among groups and the magnitude of variability within each group (**Figure 4A**). A clustering heatmap showed differences in SCFAs contents among different groups (**Figure 4B**). These results demonstrated that the levels of butyric acid, isobutyric acid, valeric acid, and 4-methylvaleric acid were significantly decreased in the BLS group compared with the control group (**Figures 4C–K**, all *P* < 0.05).

### Progression of BLS may be associated with the histidine metabolism

**Figure 5:**
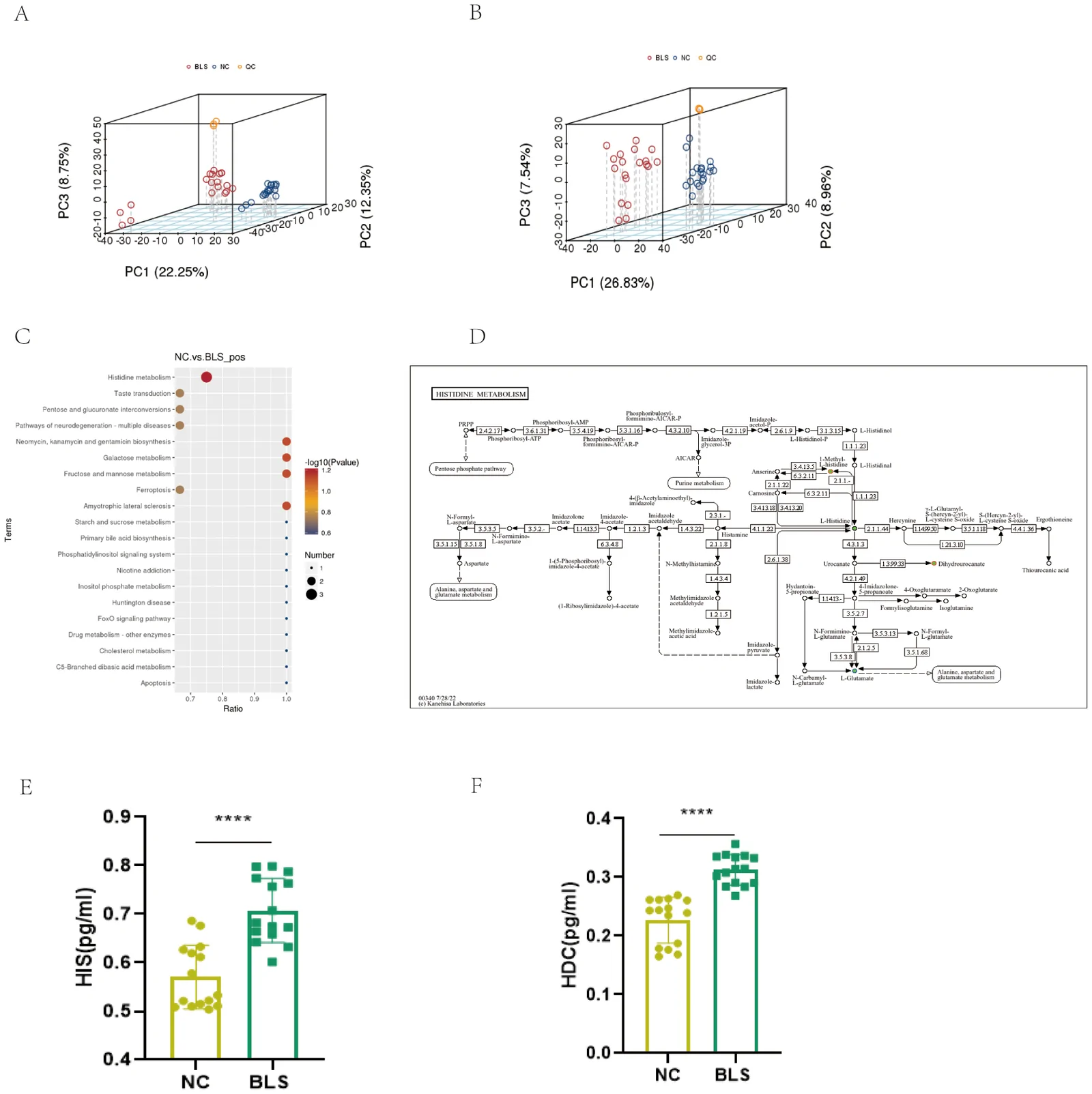
Metabolite pathway analysis of untargeted metabolomics. **(A)**. Principal component analysis in positive ion mode. **(B)**. Principal component analysis in negative ion mode. **(C)**. Histidine metabolism pathway. **(D)**. Differential metabolite pathway analysis. **(E)**. Histamine detection. **(F)**. Histidine decarboxylase (HDC) detection. Data are presented as mean ± SEM, with n = 20 per group. Significance levels are denoted as ****P < 0.0001 versus the NC group.

To investigate the potential mechanism underlying the development of brucellar spondylitis, high-throughput untargeted metabolomics was used to analyze fecal samples. We found that the metabolic differences in brucellar spondylitis were enriched in the histidine metabolism pathway. The results showed that the QC samples were closely clustered, and the smaller the difference among QC samples, the better the stability of the entire method and the higher the data quality. In addition, samples in the NC group were clearly separated from those in the BLS group, indicating that significant changes in metabolites occurred in brucellar spondylitis (**Figures 5A, B**). Subsequently, KEGG pathway analysis was performed to explore the metabolic networks *in vivo* (**Figure 5C**). The results demonstrated that the differential metabolites in brucellar spondylitis were mainly enriched in the histidine metabolism pathway compared with the control group (**Figure 5D**). Subsequently, the levels of histidine and histidine decarboxylase in the histidine metabolism pathway were detected, both of which exhibited a significantly increasing trend (**Figures 5E, F**), confirming that the progression of brucellar spondylitis may be closely associated with the histidine metabolic pathway.

### Aggravation of plasma inflammatory cytokines and LPS in patients with BLS

**Figure 6:**
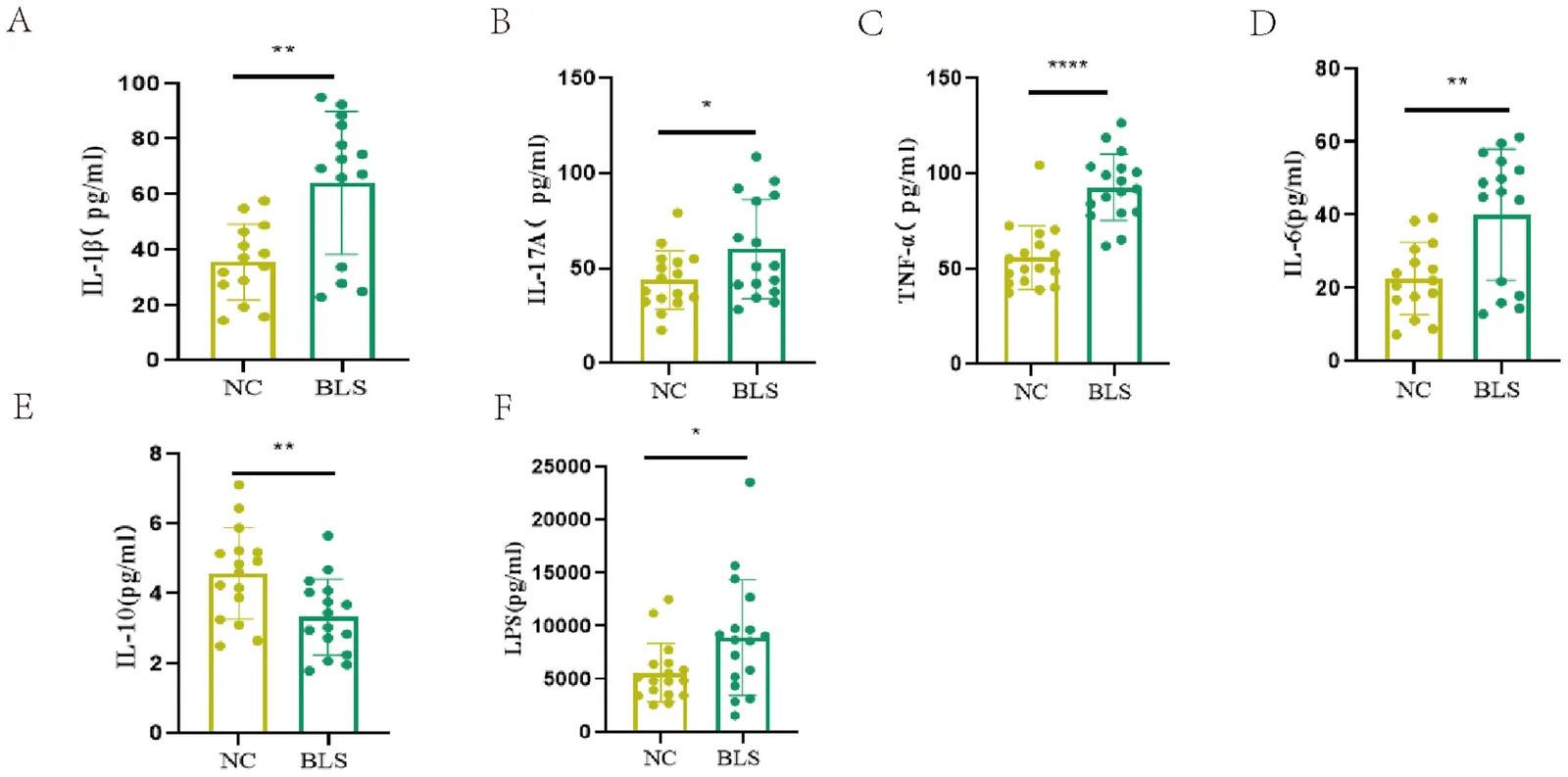
Determination of plasma inflammatory factors and LPS in Different Groups The concentrations of IL-1β **(A)**, IL-17A **(B)**, TNF-α **(C)**, IL-6 **(D)**, IL-10 **(E)**, and LPS **(F)** were determined by ELISA. Data are presented as mean ± SEM, with n = 16 per group. Significance levels are denoted as *P < 0.05, **P < 0.01, and ****P < 0.0001 versus the NC group. All experiments were performed in triplicate.

Inflammatory factors serve as a bridge connecting gut microbiota and bone damage. To determine the systemic immune-inflammatory status of patients with BLS, the levels of inflammatory factors in plasma were measured using ELISA. The results showed that, compared with the control group, TNF-α was significantly elevated, while IL-10 levels were decreased, while the levels of IL-17A, IL-1β, and IL-6 were increased in BLS group. The cytokine profile we observed is consistent with previous studies on BLS and brucellosis. Specifically, Brucella infection of osteoblasts and macrophages has been shown to directly induce the production of TNF-α, IL-1β, and IL-6 through pathways such as GM-CSF (Chen et al., 2025); IL-17 has been demonstrated to enhance local inflammation and bone remodeling in BLS (Giambartolomei et al., 2017). A recent study also confirmed that, compared with healthy controls, BS patients exhibit significantly elevated M1 macrophage-related inflammatory cytokines (IL-1β, IL-6, TNF-α) and significantly reduced anti-inflammatory cytokines (IL-4, IL-10, TGF-β) (Aribenjirigala et al., 2025). These results indicate that the reduction in the anti-inflammatory cytokine IL-10 and the elevation of pro-inflammatory cytokines including TNF-α, IL-17A, IL-1β, and IL-6 are involved in the inflammatory response in brucellar spondylitis (**Figures 6A–E**).

LPS is mainly derived from the outer membrane of Gram-negative bacteria in the intestine and normally exists only in the intestinal lumen. Compared with healthy controls, the plasma LPS level in patients with brucellar spondylitis was significantly increased, suggesting the presence of systemic endotoxemia (**Figure 6F**). These results indicate that impaired intestinal barrier function may be involved in the pathological process of brucellar spondylitis. Elevated circulating LPS can further induce excessive inflammatory response and immune activation, thereby aggravating disease activity and local spinal lesions.

### Gut dysbiosis is closely correlated with inflammation in BLS

**Figure 7:**
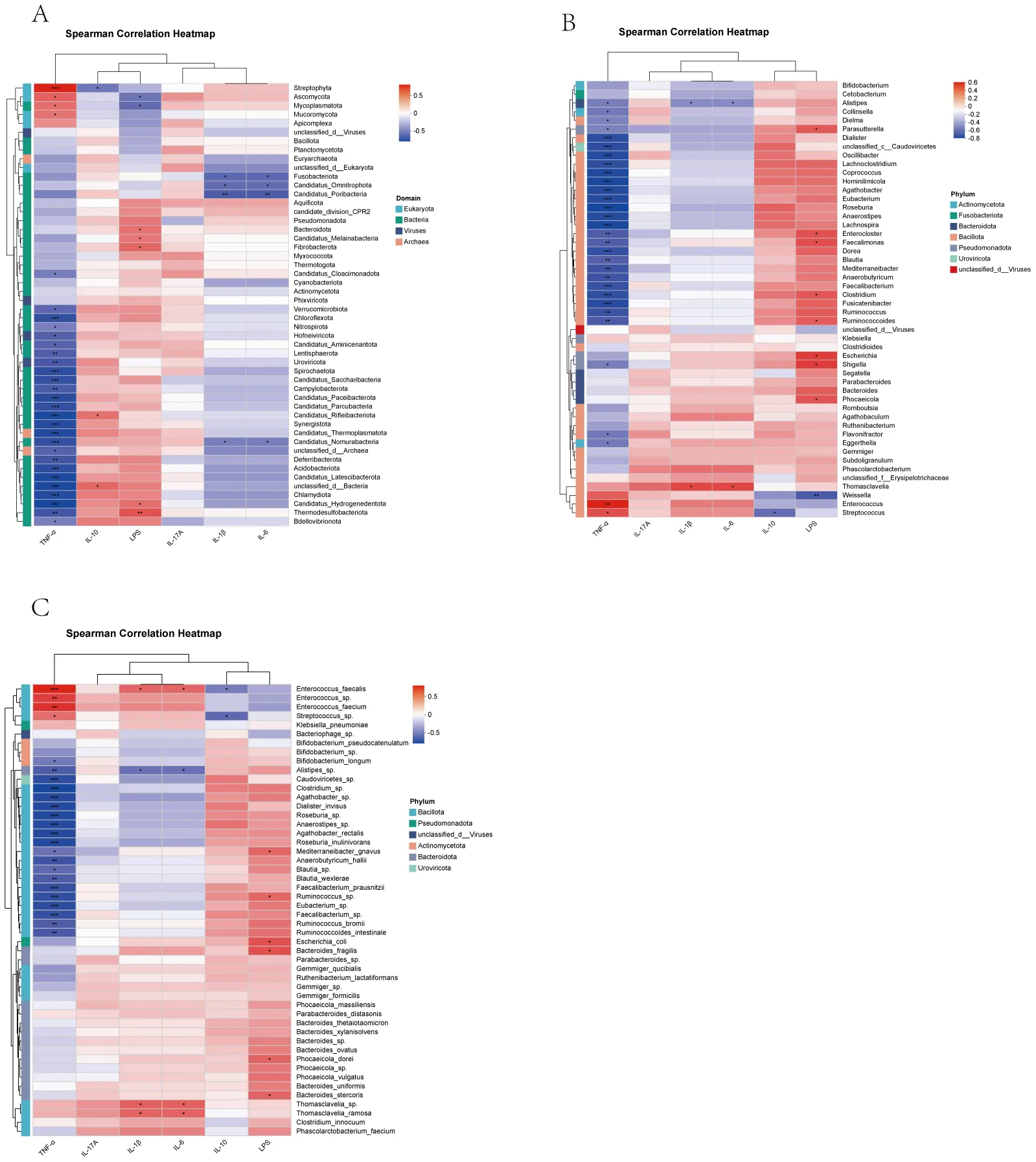
Correlation analysis between gut microbiota and inflammatory indicators. **(A)**. Correlation analysis between gut microbiota, inflammatory cytokines and LPS at the phylum level. **(B)**. Correlation analysis between gut microbiota, inflammatory factors and LPS at the genus level. **(C)**. Correlation analysis of gut microbiota with inflammatory factors and LPS at the species level. Red indicates a positive correlation, and blue indicates a negative correlation. Data are presented as mean ± SEM. Significance levels are denoted as *P < 0.05, **P < 0.01, ***P < 0.001 versus the NC group.

To determine whether there is a correlation between gut microbiota and inflammation/LPS, we further performed correlation analysis on these data. The results showed that *Bacillota* was negatively correlated with IL-10 and positively correlated with IL-6 and LPS. *Bacteroidota* was positively correlated with IL-6, IL-1β, and LPS, and negatively correlated with IL-10. *Pseudomonadota* was positively correlated with IL-6, IL-1β, and LPS (**Figure 7A**). *Cetobacterium, Alistipes, Collinsella*, *Dielma*, *Parasutterella*, *Dialister* and *unclassified_c_Caudoviricetes* were positively correlated with IL-17A, IL-1β, IL-6, and IL-10.*Oscillibacter*, *Lachnoclostridium*, *Coprococcus*, *Hominilimicola*, *Agathobacter*, *Eubacterium*, *Roseburia*, *Anaerostipes*, *Lachnospira*, *Enterocloster*, *Faecalimonas*, *Dorea*, *Blautia*, *Mediterraneibacter*, *Anaerobutyricum*, *Faecalibacterium*, and *Clostridium* were negatively correlated with IL-17A, IL-1β, IL-6, and IL-10. *Bifidobacterium* was positively correlated with IL-10. *Shigella, Parabacteroides, Bacteroides, Phocaeicola, Romboutsia, Agathobaculum, Ruthenibacterium, Flavonifractor, Eggerthella, Gemmiger, Subdoligranulum, Phascolarctobacterium, unclassified_f_Erysipelotrichaceae, T homasclavelia, Weissella, Enterococcus and Streptococcus* were negatively correlated with TNF-α and positively correlated with LPS. Collectively, these results suggest that the gut microbiota, inflammatory cytokines, and LPS are closely associated with the progression of brucellar spondylitis (**Figures 7B, C**).

## Discussion

BLS is a common and clinically severe manifestation of brucellosis, characterized by chronic low back pain, spinal deformity, and even paraplegia. These complications not only lead to physical disability and profoundly impair patients’ quality of life, limiting mobility, reducing work capacity, and causing psychological distress, but also impose a substantial socioeconomic burden through long-term care needs, productivity loss, and escalating healthcare expenditures (Spernovasilis et al., 2024). Increasing evidence indicates that the gut microbiota, as a vital metabolic and immune organ in the body, is not only involved in the occurrence and development of various chronic diseases but also regulates bone homeostasis and systemic inflammatory responses by modulating metabolic and immune networks. Gut microbiota, as a critical metabolic and immune organ, not only participates in the pathogenesis of various chronic diseases but also regulates bone homeostasis and systemic inflammation through modulating metabolic and immune networks. Studies have demonstrated that depletion of CD4^+^ T cells and CD11b^+^/GR1^+^ osteoclast precursor cells in the bone marrow, followed by restoration after transplantation, suggests that the gut microbiota regulates bone mass in mice by altering the bone immune microenvironment and affecting osteoclast-mediated bone resorption. Importantly, the osteomicrobiology framework extends beyond metabolic bone diseases to infectious conditions affecting bone. Gut microbiota dysbiosis has been shown to participate in the development and progression of osteomyelitis, and microbiota-derived metabolites influence susceptibility to bone infections such as osteomyelitis through immunomodulation and host defense mechanisms (Han et al., 2024). In the context of brucellosis, oral infection with Brucella abortus has been shown to induce significant alterations in gut microbiota composition and diversity, and these dysbiotic changes correlate with host inflammatory responses and bacterial dissemination (Rungue et al., 2021). Furthermore, gut microbiota remodeling has been demonstrated to enhance immune defense against Brucella by modulating systemic T cell responses and alleviating the inflammatory burden that drives bone erosion. In BLS, chronic inflammation and osteoclast-mediated bone resorption are central to its pathogenesis. These findings suggest that the gut microbiota could serve as a novel therapeutic target by intervening in the gut-spine axis of spinal lesions.

Regarding the structure of the gut microbiota, this study confirmed that patients with BLS exhibited significant intestinal dysbiosis. Both Alpha and Beta diversity analyses indicated that BLS patients had reduced microbial richness, a tendency toward simplified community structure, and marked remodeling of the overall microecology. At the phylum level, the relative abundances of *Bacillota* and *Pseudomonadota* were significantly decreased, while those of *Actinomycetota* and *Fusobacteriaota* were notably increased. At the genus level, beneficial SCFAs-producing bacteria including *Blautia*, *Faecalibacterium*, and *Roseburia* were significantly reduced, whereas potentially opportunistic pathogens such as *Enterococcus*, *Bifidobacterium*, and *unclassified_d_Viruses* were relatively enriched. These alterations in the gut microbiota were highly consistent with the decreased SCFAs levels, suggesting that the reduction in SCFAs-producing bacteria is the direct cause of the decline in short-chain fatty acids in BLS patients.

SCFAs, key metabolites of the gut microbiota, act as a bridge between the microbiota and the host, and play important roles in regulating immune responses and intestinal barrier function (Mukhopadhya and Louis, 2025). Butyric acid, isobutyric acid, valeric acid, and 4-methylvaleric acid were significantly decreased in the feces of BLS patients. SCFAs are key metabolites produced by gut microbiota through fermenting dietary fiber, and play important roles in maintaining intestinal barrier integrity, regulating immune responses, and inhibiting excessive inflammation. Among them, butyric acid exerts anti-inflammatory and intestinal barrier-protective effects by inhibiting HDAC activity and strengthening tight junctions of intestinal epithelial cells (He et al., 2022). The decreased SCFAs levels in BLS patients may lead to impaired intestinal barrier function and increased intestinal permeability, which in turn promote the translocation of intestinal bacteria or toxic metabolites, thereby inducing and aggravating systemic inflammatory responses (Schneider et al., 2022).

To investigate the potential mechanisms underlying the pathogenesis of BLS, high-throughput untargeted metabolomics analysis was performed on serum samples from each group, and we observed that a distinct separation of the overall metabolic profiles between the BLS group and the healthy control group. Differential metabolites were mainly enriched in the histidine metabolism pathway. The levels of histidine and histidine decarboxylase were significantly elevated in BLS patients, suggesting that abnormal histidine metabolism may be involved in the pathological process of BLS. Histidine can be converted to histamine under the catalysis of histidine decarboxylase. As an important inflammatory mediator, histamine mediates vasodilation, immune cell chemotaxis, and the release of inflammatory cytokines (Kong et al., 2026). Therefore, aberrant activation of the histidine metabolism pathway may represent an important metabolic basis for aggravating systemic inflammatory responses in BLS patients.

Anaerobic symbiotic phyla (*Candidatus_Melainabacteria*, *Chloroflexota*) and core SCFAs-producing genera (*Faecalibacterium*, *Roseburia*, *Blautia*, *Agathobacter*) as well as anti-inflammatory strains (*Faecalibacterium prausnitzii*, *Agathobacter rectalis*) were markedly depleted. These Gram-positive commensals carry complete key KOs for butyrate synthesis and histidine homeostasis; their loss leads to impaired SCFAs production and disrupted physiological histidine catabolism. Meanwhile, Gram-negative pro-inflammatory phyla (*Chlamydiota*, *Spirochaetota*, *Synergistota*) and the opportunistic pathogen *Enterococcus overproliferate*. Lacking intact butyrate synthetic machinery, *Enterococcus* is enriched with histidine decarboxylase KOs that drive excessive histamine production. Butyrate deficiency and broken intestinal barriers resulting from depleted beneficial anaerobes remodel gut anaerobic conditions, enabling the colonization of facultative anaerobic *Enterococcus*, which occupies vacant niches via proteolytic fermentation and histamine synthesis to exacerbate intestinal inflammation.

In the present study, we hypothesized that menopausal disturbance of the gut microbiota may lead to an imbalance between Th17 and Treg cell polarization. Inflammation in the bone environment is triggered by the production of anti-inflammatory and pro-inflammatory cytokines, contributing to the further progression of BLS (Hou et al., 2026). As detected in our study that plasma levels of pro-inflammatory cytokines including TNF-α, IL-17A, IL-1β, and IL-6 were significantly elevated in BLS patients, while the anti-inflammatory cytokine IL-10 was markedly decreased, indicating persistent immune activation and chronic inflammation in these patients. Meanwhile, plasma LPS levels were significantly higher in BLS patients than in healthy controls (Vargas-Caraveo and Martínez-Martínez, 2026), suggesting the presence of systemic endotoxemia. PS is mainly derived from the cell wall of intestinal Gram-negative bacteria, and its abnormal elevation in the circulation generally indicates impaired intestinal mucosal barrier function, namely the “leaky gut” syndrome. Observations in this study showed a significant increase in plasma LPS levels in the context of vertebral lesions, accompanied by impaired intestinal barrier function, linking gut microbial dysbiosis to local vertebral inflammation and bone destruction. With the proposal of the “gut-spine axis” concept, the regulatory role of gut microbiota and their metabolites in spinal disorders such as intervertebral disc degeneration and lumbar disc herniation has attracted increasing attention (Morimoto et al., 2023), while systemic immune–metabolic interactions provide a novel framework for understanding this axis. Following the translocation of large amounts of LPS into the circulation due to intestinal barrier dysfunction, the primary molecular target is Toll-like receptor 4 (TLR4) on the surface of immune cells. Upon binding of LPS to the TLR4/MD-2 complex, the intracellular adaptor proteins MyD88 and TRIF are recruited, leading to activation of the IκB kinase (IKK) complex, phosphorylation and degradation of IκBα, and ultimately nuclear translocation of the NF-κB p65 subunit. In the local vertebral microenvironment, sustained NF-κB activation directly upregulates the expression of downstream target genes, including pro-inflammatory cytokines such as tumor necrosis factor-α (TNF-α), interleukin-1β (IL-1β), and IL-6, as well as RANKL, a key factor in osteoclast differentiation. Concurrently, NF-κB acts synergistically with NFATc1 to activate osteoclast-specific genes (e.g., TRAP, Cathepsin K, and MMP-9), thereby accelerating vertebral bone resorption and destruction. This TLR4/NF-κB signaling cascade triggered by gut-derived endotoxin constitutes the core molecular pathway through which circulating LPS remotely activates local vertebral osteoclastogenesis, and the sustained activation of this pathway under systemic inflammatory conditions has been validated in various autoimmune bone diseases (Liu et al., 2025).

Beyond the direct activation of the TLR4/NF-κB signaling pathway by LPS, the migration of gut-derived immune cells to the skeletal system represents another important route of remote regulation. In spondyloarthropathies such as axial spondyloarthritis, gut microbial dysbiosis and impaired barrier function lead to aberrant activation of the mucosal immune system. Altered microbial signals, by disrupting the intestinal barrier, activate key immune pathways such as the IL-23/IL-17 axis, promoting the activation of innate-like lymphocytes including mucosal-associated invariant T (MAIT) cells, γδ T cells, and ILC3s. More direct evidence demonstrates that bacterial DNA can co-localize with inflammatory macrophages in the gut lamina propria, peri-articular bone marrow, entheses, and spleen, and these myeloid cells carrying gut-derived bacterial components can migrate through the circulation to bone and joint sites, where they locally drive inflammatory responses and promote osteoclastogenesis (Li et al., 2026). This “gut immune cell–bone” trafficking pathway provides a cellular-level mechanistic explanation for the link between circulating LPS and local vertebral inflammation. Furthermore, systemic alterations in gut microbial metabolites, particularly the metabolic interplay between reduced SCFAs and excessive histamine production, constitute another dimension of the remote pro-inflammatory effects of circulating LPS. SCFAs and histamine are two key classes of metabolites produced by the gut microbiota, and they exhibit a complex reciprocal regulatory relationship. Studies have shown that therapeutic supplementation with the SCFAs propionate expands local intestinal histamine concentrations and drives the resolution of inflammation via the histamine-H3R axis in arthritis models, defining a “gut–central nervous system–joint” metabolic pathway (Dürholz et al., 2025). In the state of gut microbial dysbiosis, reduced SCFAs production weakens the maintenance of intestinal epithelial integrity and immune homeostasis, while histidine decarboxylase-expressing bacteria convert large amounts of histidine into histamine, leading to excessive histamine production. Histamine interacts with other microbial metabolites, including SCFAs and tryptophan derivatives, forming a complex metabolic–inflammatory network that can amplify fibroblast-like synoviocyte activation, osteoclastogenesis, and chronic inflammation (41).

In summary, the findings of this study can be understood within the following integrated framework: after gastrointestinal infection, Brucella triggers gut dysbiosis, which in turn impairs intestinal barrier function; the resulting barrier dysfunction enables systemic translocation of lipopolysaccharide (LPS), which remotely activates vertebral osteoclast differentiation via the TLR4/NF-κB signaling pathway. Concurrently, gut-derived activated immune cells carrying bacterial components migrate to the bone microenvironment, where they locally provoke inflammatory responses. Meanwhile, a metabolic imbalance between reduced SCFAs and excessive histamine production further exacerbates osteoclastogenesis and bone destruction. These three pathways intertwine and act synergistically, together constituting a complete molecular bridge between circulating LPS and local vertebral inflammation. Future studies should integrate animal experiments and clinical interventions to validate the adjunctive therapeutic value of targeted gut microbiota modulation (e.g., supplementation with SCFAs-producing bacteria, prebiotics, or fecal microbiota transplantation) for BLS, and to further elucidate the specific mechanisms by which histidine metabolism participates in immune regulation in BLS.

This study also has several limitations. First, the sample size was relatively small, and the conclusions need to be verified in a larger population. Second, the cross-sectional design limited the inference of causal relationships. In addition, fecal microbiota and metabolites only reflect the local intestinal status, and their direct association with local spinal lesions requires further investigation. Future studies should combine multi-omics techniques and dynamic follow-up to further reveal the regulatory mechanism of the gut-spine axis in BLS.

## Conclusion

BLS is associated with gut microbiota dysbiosis and alterations in microbial metabolites, which may be linked to inflammatory responses and histamine metabolism. The differential microbial taxa identified in this study could be developed into a stool-based non-invasive diagnostic panel to facilitate early differentiation of BLS from other spinal disorders. Furthermore, restoring gut microbial balance through probiotic supplementation or dietary modulation may represent a promising adjunctive strategy to enhance the efficacy of standard antibiotic therapy and reduce disease recurrence.

## Data Availability

The datasets presented in this study can be found in online repositories. The names of the repository/repositories and accession number(s) can be found in the article/supplementary material.
